# Predictive long-range allele-specific mapping of regulatory variants and target transcripts

**DOI:** 10.1371/journal.pone.0175768

**Published:** 2017-04-13

**Authors:** Kibaick Lee, Seulkee Lee, Hyoeun Bang, Jung Kyoon Choi

**Affiliations:** 1 Department of Bio and Brain Engineering, KAIST, Daejeon, Republic of Korea; 2 Graduate School of Medical Science and Engineering, KAIST, Daejeon, Republic of Korea; Pohang University of Science and Technology, REPUBLIC OF KOREA

## Abstract

Genome-wide association studies (GWASs) have identified a large number of noncoding associations, calling for systematic mapping to causal regulatory variants and their distal target genes. A widely used method, quantitative trait loci (QTL) mapping for chromatin or expression traits, suffers from sample-to-sample experimental variation and *trans*-acting or environmental effects. Instead, alleles at heterozygous loci can be compared within a sample, thereby controlling for those confounding factors. Here we introduce a method for chromatin structure-based allele-specific pairing of regulatory variants and target transcripts. With phased genotypes, much of allele-specific expression could be explained by paired allelic *cis*-regulation across a long range. This approach showed approximately two times greater sensitivity than QTL mapping. There are cases in which allele imbalance cannot be tested because heterozygotes are not available among reference samples. Therefore, we employed a machine learning method to predict missing positive cases based on various features shared by observed allele-specific pairs. We showed that only 10 reference samples are sufficient to achieve high prediction accuracy with a low sampling variation. In conclusion, our method enables highly sensitive fine mapping and target identification for trait-associated variants based on a small number of reference samples.

## Introduction

Most disease associations discovered by genome-wide association studies (GWASs) are distant from coding genes. It was shown that these noncoding variants are concentrated in regulatory DNA marked by DNase I hypersensitivity[1] or histone modifications[2]. This enables epigenetic fine mapping of noncoding GWAS single-nucleotide polymorphisms (SNPs)[3]. However, overlapping itself does not mean functionality. In this regard, quantitative trait loci (QTL) mapping and more recently, allele-specific analyses, are used to test the functional differentiation of different alleles in terms of chromatin accessibility[4,5], histone modification levels[6–11], or transcription factor binding[12].

Additional methods and data are required to map these functional regulatory variants to their target genes. Expression QTL (eQTL) analysis has been commonly used for the variant-gene mapping[13–16]. However, eQTL mapping hinges on statistical association, which may fail to detect the direct regulatory relationships because primary target genes usually trigger a cascade of downstream regulatory reactions. To identify direct physical targets, it is essential to determine three-dimensional chromatin structure[17,18]. Chromatin interactome is expected to provide critical resources for understanding the action mechanism of disease variants as illustrated in obesity[19,20].

QTL mapping for either chromatin or expression traits may suffer limited sensitivity because of technical variation and *trans*-acting effects. Because of sample-to-sample experimental bias, for example due to different read depths or other unspecified batch effects, true biological variation is often buried in confounding technical noise. The effect of *trans*-acting mechanisms can be illustrated by negative-feedback control that cancels out variability across samples with different *cis*-regulatory genotypes[21]. In contrast, allele-specific analyses leverage the intrinsic power of using a within-sample control, which enables elimination of technical, environmental, or *trans*-acting influences. Therefore, this approach should confer greater sensitivity in uncovering the direct influence of *cis*-regulatory variation[21].

To overcome the limited sensitivity, QTL mapping requires a large cohort of samples to increase statistical power. In contrast to QTL mapping, allelic analyses are not hampered largely by small sample size, insofar as there is at least one heterozygous sample with a sufficient read depth for the given locus. However, there is limitation in coverage (i.e., how many loci can be tested) especially for less frequent variants. The same problem applies for QTL mapping. A powerful workaround may be the employment of machine learning. Diverse features of the identified variants can be learned and used to predict functional variants that were not testable using given samples. Here we first perform allele-specific, long-range mapping of *cis*-regulatory elements and target transcripts, and then apply machine learning for the identified pairs.

## Results

### Long-range allelic mapping of immune GWAS results

We collected data of chromatin immunoprecipitation-sequencing (ChIP-seq) for histone modifications (including H3K27ac and H3K4me1), RNA-sequencing (RNA-seq), and phased genotyping on 100 genetically different lymphoblastoid cell lines[6–9,22] (S1 Table). This data collection served as a reference genetic panel for the allelic analyses of variants in question. As a set of test variants, we obtained 2,351 SNPs associated with autoimmune diseases, allergic diseases, inflammation-related diseases, and laboratory results for immune cells (S2 Table) from the GWAS catalogue. Additionally, 7 reference chromatin interactome datasets in immune-related cells were collected (S3 Table).

**Fig 1:**
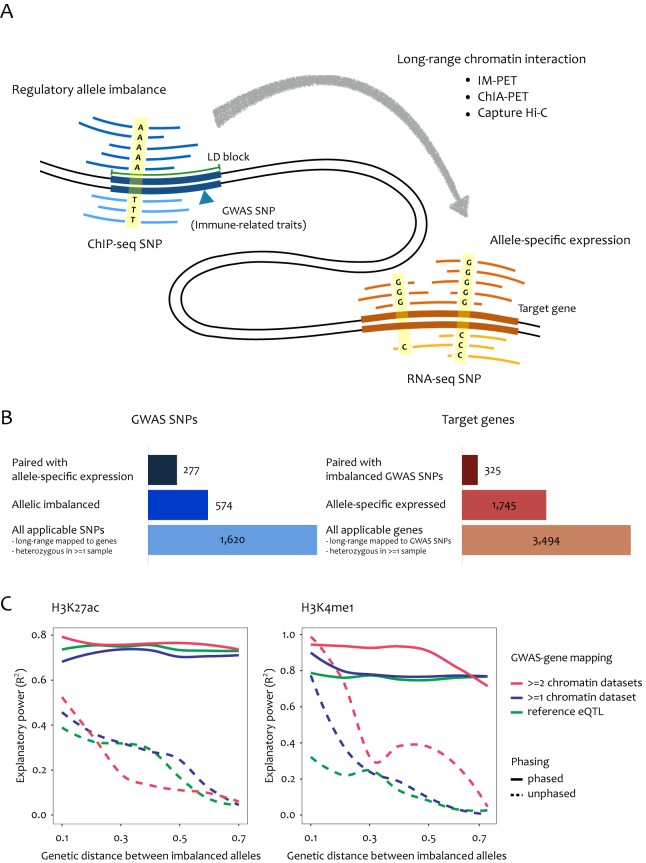
Summary of long-range allele-specific mapping. (A) Schematic view of our method. We first search for SNPs causing allele imbalance in ChIP-seq reads within the LD block of a given GWAS SNP. We then search for allele-specific expression of transcripts connected via chromatin interaction. The imbalance SNPs are paired when the regulatory direction of their phased genotypes matches with each other (i.e., when the major regulatory allele and major transcript allele are on the same chromosome for the activating histone marks). (B) The number of GWAS SNPs (upper) and target genes (lower) at the beginning, after allele imbalance analysis, and after long-range pairing (from bottom to top). The applicable SNPs and genes were defined as being heterozygous in at least one sample and possessing chromatin interaction. (C) Explanatory power indicating the extent to which allele-specific expression is explained by allele-specific *cis*-regulation. Linear regression was performed where the RNA-seq allele ratio was regressed on the paired ChIP-seq allele ratio. Pairing was done by chromatin interaction versus eQTL mapping with phased versus unphased genotypes.

Fig 1A summarizes our analysis scheme. We first examined allelic imbalance in ChIP-seq reads of H3K27ac, H3K4me1, H3K4me3, H3K27me3, and H3K36me3 for SNPs in linkage disequilibrium (LD) with the GWAS SNPs. By using the chromatin interactomes in immune-related cells (S3 Table), ChIP-seq peaks were mapped to their target transcripts. Of 2,351 GWAS SNPs, 1,620 were in LD with at least one SNP that was heterozygous in at least one sample while residing in any long-range mapped ChIP-seq peaks. These target transcripts were also searched for allele imbalance in RNA-seq. We performed meta-analysis for multiple heterozygous samples. Then we paired ChIP-seq imbalance and RNA-seq imbalance by considering the regulatory directionality of phased SNPs (i.e., paired when the major regulatory allele and major transcript allele are on the same chromosome for activating histone marks, and the opposite for repression marks). The overall statistics are given in Fig 1B. Finally, 277 GWAS SNPs and 325 transcripts were paired. Gene Ontology analysis showed significant enrichment for immune-related function (S4 Table).

We examined the degree to which regulatory allele imbalance was reflected in paired allele-specific expression. To this end, the transcript allele ratios were regressed on the paired ChIP-seq allele ratios for each histone mark, and the explanatory power of the linear regression model was obtained. Here we focused on H3K27ac and H3K4me1. The explanatory power of linear regression for the imbalance scores was consistently high across varying distances between the paired SNPs (solid lines in Fig 1C). However, without phasing, the allelic regulatory association maintained only within a short range (dotted line in Fig 1C) because the regulatory direction cannot be matched using the reference genome for distant SNPs that are not in LD. This indicates that genotype phasing is essential to map long-range regulatory associations. For comparison, we used reference eQTL data in lymphoblastoid cells in place of chromatin interactomes, and found a similar or lower performance (green lines in Fig 1C). The permutations of chromatin interactions reduced the explanatory power (grey curves in S1 Fig).

### Comparison of allelic mapping and QTL mapping

**Fig 2:**
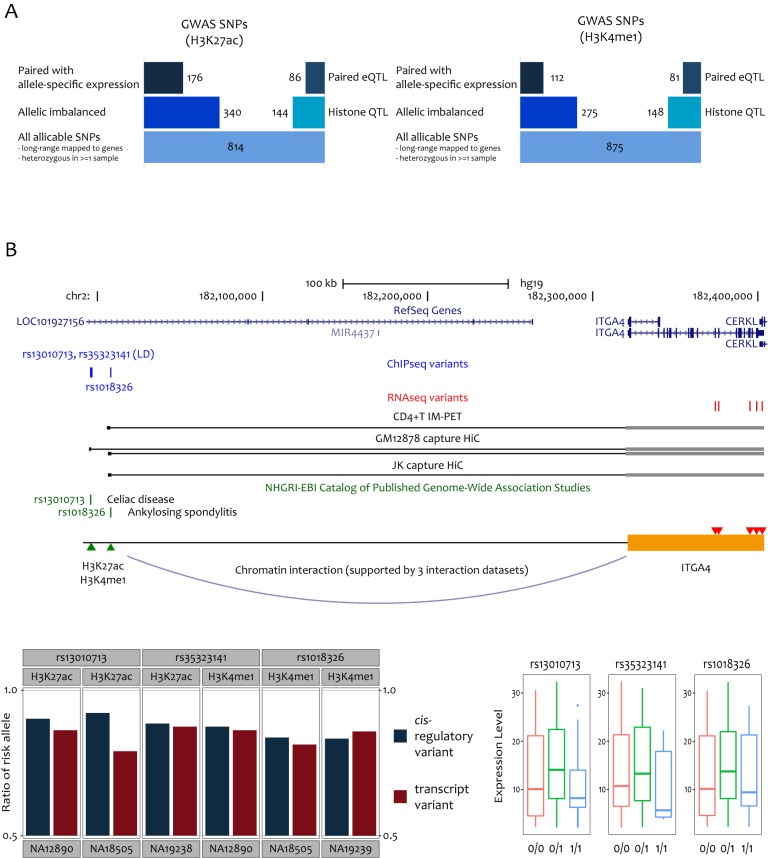
(A) Comparison of sensitivity between allelic mapping versus eQTL mapping. The number of GWAS SNPs with ChIP-seq imbalance versus that of GWAS SNPs that are histone QTLs (middle row), and the number of the imbalanced GWAS SNPs with paired allele-specific expression versus that of the histone QTLs that are eQTLs for paired genes (top row). For both allelic mapping and eQTL mapping, an unadjusted P < 0.05 was used as a threshold. (B) Allelic mapping for ITGA4. Three SNPs (blue) showed allele imbalance in ChIP-seq reads (blue bar graphs) with bias towards the disease risk allele. These SNPs were connected to ITGA4 as indicated by three different chromatin interactome datasets (black lines). The RNA-seq variants (red) showed allele-specific expression (red bar graphs) in the same direction as the ChIP-seq variants. eQTL mapping failed to detect association (boxplots).

We sought to compare the sensitivity of allelic mapping and QTL mapping. Based on the same reference panel samples used for our allelic mapping, we first identified histone QTLs (hQTLs), and then searched them for eQTLs by associating them with the expression level of genes connected via chromatin interaction. For fair comparison, we started with the same sets of 814 and 875 GWAS SNPs for which at least one LD SNP was heterozygous in at least one sample and that resided in H3K27ac and H3K4m1 regions having chromatin interactions (Fig 2A). Because H3K27ac and H3K4me1 are activation marks, we selected the cases in which histone modification and gene expression changed in the same direction according to the underlying genotype. With the same statistical confidence (unadjusted P < 0.05), the allelic method identified approximately two-fold greater number of causal *cis*-regulatory variants and paired transcripts than the QTL approach (Fig 2A). This underscores that fact that allelic mapping can provide higher sensitivity than QTL mapping.

As for H3K27ac, there were 176 GWAS SNPs that were identified by our allelic analyses. Among them, 35 SNPs (20%) were also identified by the histone QTL-eQTL methods. Regarding H3K4me1, 21 out of 112 SNPs (19%) were supported by QTL mapping. However, there were more cases in which only allelic mapping was able to detect. For example, ITGA4, a therapeutic target for multiple sclerosis[23] and Crohn’s disease[24], was mapped to other autoimmune diseases through our allele-specific analysis (Fig 2B). The risk alleles were overrepresented in the ChIP-seq reads and were on the same chromosome as the alleles overrepresented in the RNA-seq reads. This pattern was consistent for the different disease alleles, indicating that ITGA4 overexpression may be generally associated with an increased risk of autoimmune diseases. This is in good agreement with the role of this gene in promoting adhesion and migration during autoimmune responses and with the therapeutic effects of its antagonist[23,24]. However, it was not possible to identify this gene through QTL mapping (Fig 2B).

Another example was RASSF5. This gene was recently shown to negatively control lymphocyte proliferation and prevents autoimmunity[25]. Indeed, the risk alleles associated with certain autoimmune diseases were underrepresented in the ChIP-seq reads and were on the same chromosome as the alleles that were underrepresented in the RNA-seq reads (S2 Fig). Therefore, one can hypothesize that these alleles increase the disease risk by inhibiting RASSF5 gene transcription. As shown in the figure, eQTL mapping failed to detect the association despite multiple chromatin interactome datasets supporting physical enhancer-promoter interaction.

Previous QTL studies commonly reported that many of regulatory variants associated with histone modification or TF binding were not associated with gene expression variability[6–12]. In our analysis, ~60% of hQTLs were eQTLs for genes paired through chromatin interactions. The corresponding percentage was slightly lower for the allele-specific pairs (i.e., 40~50% of regulatory imbalance was paired with allele-specific expression). Even with improved sensitivity owing to inherent control for confounding factors, allele-specific expression may be relatively more difficult to detect compared to regulatory imbalance because exonic variants should be less frequent than noncoding variants.

### Predictive allelic mapping

Coverage limitations can be imposed not only by the absence of transcript variants but also due to the unavailability of *cis*-regulatory or exonic heterozygotes especially when sample size is small. Therefore, we employed a machine learning method that predicts missing positive pairs whose allelic imbalance cannot be directly tested. To apply machine learning, we first identified testable cases by checking whether we could assess allelic imbalance thanks to the presence of heterozygous loci with sufficient read depth in each sample. Some of the testable pairs passed our imbalance tests and were used as true cases for machine learning. The testable pairs that failed to pass the imbalance tests, in other words, the cases in which *cis*-regulatory regions or transcripts show no allelic imbalance, were used as false cases. We trained Random Forest to learn 259 features (S5 Table) of the allele-specific pairs (true cases) against the features of the false cases as the control set. Area under the curve (AUC) was measured by using testing samples on the basis of 5-fold cross validation. High prediction performance was obtained as shown by the red receiver operating characteristic (ROC) curves in Fig 2B. Proper learning was failed when the features were randomly assigned to each pair (grey ROC curves in S3 Fig), indicating that the observed allele-specific pairs indeed share certain features that distinguish them from the non-functional variants in the control set.

**Fig 3:**
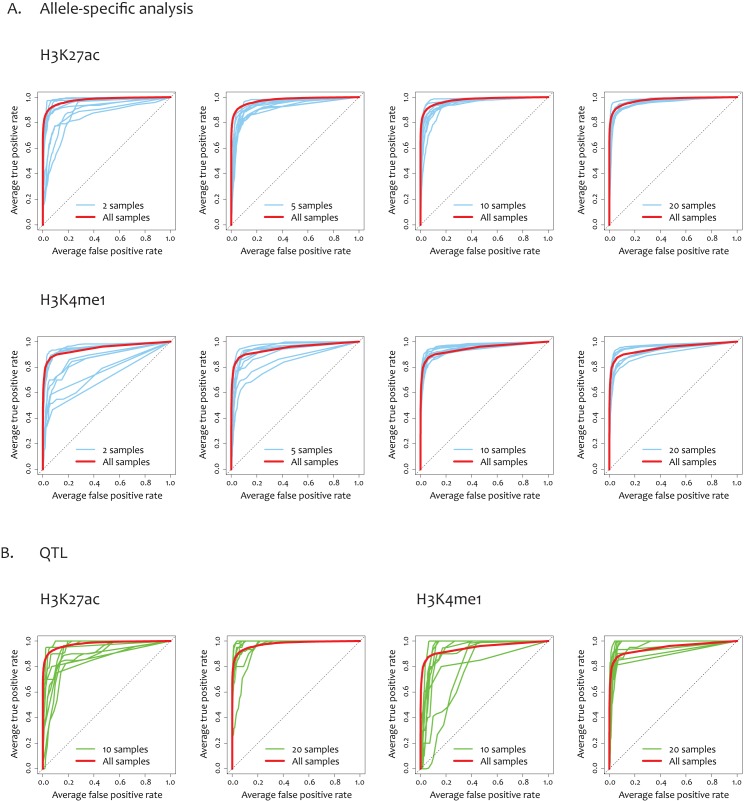
(A) ROC curves representing the accuracy of predictive allelic mapping. Among all pairs of ChIP-seq and RNA-seq variants connected via chromatin interaction, we collected true cases (pairs showing allele imbalance in both ChIP-seq and RNA-seq) and subjected them to Random Forest training. ROC curves were generated based on 5-fold cross-validation with each validation process based on two Random Forest models. The red curves represent performance achieved with the entire set of samples (100 samples for H3K27ac and 59 for H3K4me1). The blue curves show performance with a subset of samples. The sampling was repeated 10 times to estimate variation in performance. (B) ROC curves indicating the accuracy of predictive QTL mapping. Among all pairs of ChIP-seq and RNA-seq variants connected via chromatin interaction, we collected true cases (histone QTLs that are eQTLs for the paired genes) and subjected them to Random Forest training. The red curves represent performance achieved with the entire set of samples (100 samples for H3K27ac and 59 for H3K4me1). The green curves show performance with a subset of samples. The sampling was repeated 10 times to estimate variation in performance.

Random Forest was feasible only with 2 samples, but its performance varied depending on which samples were used (blue ROC curves in Fig 3A). Sampling bias in performance was significantly lower with 5 samples and reached a robust level with 10 samples. This indicates that 10 samples with natural genetic variation have a sufficient number of testable (heterozygous) cases for proper machine learning. The average number of the true cases per sample was 53 for H3K27ac and 78 for H3K4me1. Therefore, with 10 samples, more than 500 true cases could be used for training on average. The number of the false cases was 843 for H3K27ac and 1291 for H3K4me1. When we repeated similar procedures for hQTL-eQTL pairs, considerable variability existed among 10 sample-based prediction results (green ROC curves in Fig 3B).

**Fig 4:**
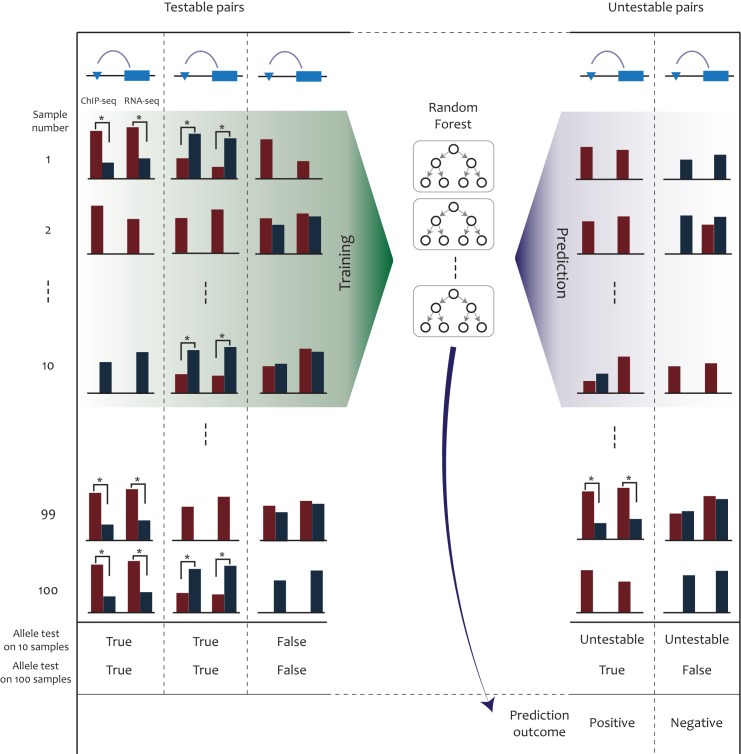
Schematic of predictive allelic mapping using a small number of samples. In this illustration, the 10 samples in the first 10 rows are used for training and prediction. Over the whole-genome, testable and untestable pairs of regulatory regions and transcripts are collected. The red and blue bars represent the number of allele-specific ChIP-seq or RNA-seq reads. Heterozygotes should have the two bars simultaneously whereas homozygotes should have only one. The prediction outcome is validated by performing allelic tests using additional samples. In this illustration, the remaining 90 samples were used for this purpose. For example, one of the untestable pairs is called positive by Random Forest, and indeed shows allele imbalance in one sample heterozygous for the regulatory region and transcript.

By using the 10-sample Random Forest models, we sought to test the utility of our method in predicting missing positive cases. To this end, we selected untestable cases, in which we were not able to assess allelic imbalance due to the absence of heterozygotes (Fig 4). The sampling was repeated 10 times. On average, our Random Forest classifier rescued ~ 89 H3K27ac pairs and ~ 50 H3K4me1 pairs that were not available for allele-specific tests using the given 10 samples. We examined how many of them could be confirmed to be actually allele-specific (Fig 4). For H3K4me1, the number of tested pairs was too low because the entire set consisted of 59 samples. Regarding H3K27ac for which there were 100 samples, allele specificity for > 87% of the predicted pairs was corroborated when they were tested using the whole set of available samples (Table 1). In other words, the corroborated cases means that they were untestable on the initial 10 samples, but were predicted to be positive and turned out to be allele-specific when tested using the 100 panel samples.

**Table 1:** Validation of predictive allelic mapping.

Sampling number	Number of predicted pairs	Number of tested pairs	Allele-specific pairs (percentage)
1	89	72	70 (97.2%)
2	51	26	25 (96.2%)
3	56	24	21 (87.5%)
4	82	60	54 (90%)
5	37	13	13 (100%)
6	54	21	18 (85.7%)
7	62	24	19 (79.2%)
8	52	25	17 (68%)
9	47	19	16 (84.2%)
10	45	30	27 (90%)

## Discussion

For a given set of trait-associated variants (tag SNPs), our method enables the identification of causal regulatory variants in LD and their functional target genes. This task requires a reference panel consisting of a small number of samples for which matched epigenome, transcriptome, and phased genotypes are available. In addition, a reference chromatin interactome dataset is needed to link regulatory variants and target genes. Instead, large-scale reference eQTL data can serve the same purpose. For example, the GTEx project[13] provides genetic associations in different tissues. The reference panel and chromatin interactome data should be based on the tissue relevant to the trait in question. In our model, we used immune-related traits and reference data in lymphoblastoid cell lines and other immune or blood cells. It is also important to prepare feature sets related to the given trait for predictive allelic mapping using machine learning. In our analysis, we used Gene Ontology terms such as immune process and inflammatory response. For epigenomics features, we utilised the Blueprint Project data in distincit types of haematopoietic cells. All the reference data, feature sets, and training outcomes are provided in our software (see Materials and Methods). Users can search for causal variants and functional target genes for their SNPs associated with immune-related traits. Direct allelic mapping can be first performed and for untestable cases, Random Forest will provide predicted functional pairs for each input SNP.

Similar attempts can be made through QTL mapping. However, this requires a large number of samples. The accuracy of QTL mapping increases in proportion to the number of samples used. While reference eQTL data have been made available in many tissues, hQTL or other chromatin QTL mapping has been performed only in lymphoblastoid cells. With eQTL data alone, causal variants and their direct, physical target genes cannot be mapped. Moreover, there is inherent limitation that undermines the sensitivity of association detection. Our results show that only ten samples enable highly sensitive detection of allele specificity. This approach can be extended to cover different traits. For example, obesity associations can be dissected by examining a small number of reference data based on genetically different adipose tissues. In conclusion, our method is expected to assist in the annotation of a large number of trait-associated variants residing noncoding regions of the genome.

## Materials and methods

### Reference genetic panel

We collected data of ChIP-seq for histone modifications (including H3K27ac and H3K4me1), RNA-seq, and phased genotyping in 100 genetically different lymphoblastoid cell lines[6–9,22]. The first three related datasets[6–8] included RNA-seq and five types of ChIP-seq (H3K27ac, H3K27me3, H3K36me3, H3K4me1 and H3K4me3) of 24 samples. These data were available under accession numbers E-MTAB-1883 and E-MTAB-1884 at ArrayExpress (http://www.ebi.ac.uk/arrayexpress/) and under accession numbers GSE47991, GSE19480, and GSE50893 at Gene Expression Omnibus (GEO) (http://www.ncbi.nlm.nih.gov/geo/). From another study[9], H3K27ac ChIP-seq data on 57 YRI samples were collected. The corresponding data and matched expression data[16] were available at the GEO with accession number GSE58852 and GSE19480, respectively. Additionally, we collected RNA-seq and three types of ChIP-seq (H3K27ac, H3K4me1, and H3K4me3) data on 47 CEU samples[22]. The RNA-seq data were available under accession number E-MTAB-3656 and histone ChIP-seq data were under E-MTAB-3657 at ArrayExpress. DNase-sequencing data[16] were not used because of low read depths. Collected data are summarized in S1 Table.

### Mapping and variant calling

The bam files of the collected raw data had been aligned to different releases of the reference genome. Thus, we re-mapped the data to GRCh37/hg19[26,27]. The RNA-seq and ChIP-seq raw data were mapped by using TopHat2[28] and BWA mapper[29], respectively. Each remapped bam file was then subjected to variant calling according to the “GATK Best Practices” workflow (https://www.broadinstitute.org/gatk/guide/best-practices) by using the Picard tool[30] and GATK tool[31]. In the variant filtration process, we discarded variants with < 2.0 QualByDepth (QD: QUAL score normalized by allele depth) or > 30.0 Phred-scaled P value.

### GWAS SNPs and LD expansion

A total of 2,351 GWAS SNPs spanning 51 immune-related diseases and traits (S2 Table) were retrieved from the National Human Genome Research Institute GWAS Catalog[32]. We conducted an LD expansion of these GWAS SNPs using Haploview[33]. Instead of the built-in HapMap genotype data, genotype information from the 1000 Genomes Project[34] (http://www.1000genomes.org/) phase 3 was referenced for LD calculation. For each human subpopulation (CEU, YRI, CHB and JPT), GAB blocks and GAM blocks, by the algorithm of Gabriel et al.[35] and the four gamete rule, respectively, were constructed. The two blocks of the same ethnicity were then merged. We searched for SNPs residing in the same block as its associated GWAS SNP commonly in all the populations (CEU, YRI, CHB, and JPT). After all, we were left with 19,584 GWAS LD SNPs.

### Detection of allele imbalance

Among variants that passed all filtering processes, heterozygous SNPs were selected. Sufficient sequencing depth is required for accurate allele imbalance testing[21]. We thus checked the allelic depth (AD according to the VCF v4.1 specification[36]) of the filtered heterozygous SNPs and chose those with the sum of reference and alternative allele depths > 8 and with the imbalance ratio between 0.15 and 0.85, as previously suggested[37]. In the cases in which the same sample was analyzed with RNA-seq or ChIP-seq for the same histone mark by two or more studies (i.e., there are two or more citation numbers in a single entry in S1 Table), the average allelic depth and ratio were considered by dividing by the number of studies. As for the detection of allelic imbalance, we performed the binomial test with p = 0.5[21] and retained the cases with P < 0.05. For the loci that were tested in multiple samples, we performed meta-analysis by combining the P values based on the Fisher’s method[38]. The χ^2^ P = 0.05 was used as a threshold for allelic imbalance across multiple samples.

### Target gene mapping, phasing, and allelic pairing

RNA-seq variants were assigned to their respective gene using the transcript location data provided by the hg19 version of RefSeq. We employed seven chromatin interactome datasets (listed in S3 Table) that were derived from different technologies encompassing chromatin interaction analysis by paired-end tag (ChIA-PET) sequencing, capture Hi-C, and integrated methods for predicting enhancer targets (IM-PET) in lymphoblastoid, K562, Jurkat, and CD4 T cells. We only used intrachromosomal interactions. The reference and alternative alleles were defined based on the unphased reference genome GRCh37/hg19. Therefore, the RNA-seq SNP and ChIP-seq SNP mapped via chromatin interaction are more likely to be unphased as they become distant from each other. Therefore, we flipped the allele ratios when the two SNPs were on different chromosomes in the phased genotype data of the 1000 Genomes Project[34] phase 3. SNPs without 1000 Genomes genotype were excluded from further analyses. Furthermore, we matched the regulatory direction (i.e., activation or repression) of the phased and mapped variants on the RNA and *cis*-regulatory region. H3K27ac, H3K36me3, H3K4me1, and H3K4me3 were regarded as activating marks while H3K27me3 was regarded as a repressive mark. We paired ChIP-seq imbalance and RNA-seq imbalance only when the major regulatory allele and major transcript allele were on the same chromosome for the activating histone marks, and the opposite for the repression mark. For functional analysis of the paired genes, we ran WEB-based GEne SeT AnaLysis Toolkit (WebGestalt)[39] and obtained P values based on the hypergeometric enrichment test and multiple testing adjustment[40].

### Explanatory power of regulatory imbalance

We wanted to test the extent to which allele-specific expression is explained by allele-specific *cis*-regulation. The transcript allele ratios were regressed on the paired *cis*-regulatory allele ratios for each histone mark, and the explanatory power of the linear regression model was obtained as *R*^2^. There were cases in which multiple *cis*-regulatory regions were mapped to a single transcript. In these cases, we considered the multiple pairs independently. The explanatory power was plotted according to the genetic distance between the two variants, 1−|*r*|, where *r* is the Pearson coefficient of correlation measuring linkage disequilibrium between the two loci. The *r* value was obtained by using the 1000 Genomes Project[34] phase 3 data. The average *R*^2^ was computed for all pairs within a given genetic distance. For comparison with the chromatin interactome data, we used reference eQTL data for mapping cis-regulatory variants to their target genes. A total of 358,199 and 478,204 significant eQTL-gene pairs in whole blood and lymphoblastoid cells[13,14], respectively, were used to replace the chromatin interactions.

### A model for predictive allelic mapping

We chose Random Forest[41] for our predictive allelic mapping. A cis-regulatory variant and target gene pair that was linked by chromatin interaction was used as a unit of evaluation. The true set for training consisted of paired cis-variants and target genes both showing allelic imbalance in the given samples. As a control set, we collected the cases in which either cis-regulatory region or target gene shows no allelic imbalance even when heterozygotes are available among the given samples. Because different histone marks lead to different true and control sets, we trained our Random Forest classifier for each histone modification separately. As for H3K27ac, there were 1,034 true pairs and 8,450 control pairs. For H3K4me1, there were 475 and 9,325 true and control, respectively. We selected features regarding the disease associated with the GWAS SNP, mapped target gene, transcription factor that is predicted to bind the cis-regulatory variant, and epigenetic marks at the distal and proximal regulatory region of the gene (S5 Table). Epigenomic feature data were retrieved from the Blueprint Project (http://www.blueprint-epigenome.eu/). We chose cell lines related with inflammation processes, including CD4+ T cells, CD8+ T cells, macrophages, monocytes, neutrophils, NK/T cells, and B cells. In addition, GM12878 and K562 data were obtained from the UCSC Genome Browser. All available histone ChIP-seq data were used. All the histone ChIP-seq data were in the narrow-peak bed file format. We assigned 1 or 0 for each ChIP-seq feature. For distal cis-regulatory regions, we assigned 1 if ChIP-seq peaks covered the variant of interest. For the promoter region of target genes, we assigned 1 when the ChIP-seq peak covered at least half of the promoter (1.5 kb upstream ~ 0.5 kb downstream of the transcription start site (TSS)). For the features of target genes, we determined whether each gene belonged to a specific GO term. We chose GO terms related to immune process and inflammatory response. The distance from the cis-regulatory variant to the target gene was defined based on the TSS of the target gene. The affinity of transcription factor binding at the distal cis-regulatory variant was estimated by FIMO[42]. ROC plots were drawn based on a 5-fold cross validation with two repetitions each time, resulting in 10 different classifiers. A total of 1,000 decision trees were used for each Random Forest classifier. Each classifier determined whether the pair of the given *cis*-regulatory variant and its linked gene shares similar features as those with observed allelic imbalance. We used an R package named randomForest[43]. The default mtry (square root of the number of variables) and node size (minimum size of terminal node = 1 and maximum number of terminal nodes trees in the forest can have = NULL) were used. We also trained Random Forest using the true and control datasets identified based on QTL mapping. We identified hQTLs (H3K27ac and H3K4me1) and eQTLs based on the same dataset as used for allelic mapping. Linear regression models were fit between the genotypes and the quantified measures of histone ChIP-seq or RNA-seq. Using mapped BAM file from previous dataset, we called ChIP-seq peak and calculated peak intensity by HOMER[44]. From the linear regression, we obtained the effect size and P value for each tested association. hQTLs for H3K27ac and H3K4me1 were identified and linked to their target genes via chromatin interactions. The true set consisted of hQTLs that were eQTL of their connected target gene. The control set was composed of hQTLs that were not eQTL of their connected target gene. In a similar manner as our predictive allelic mapping, the whole set and subsets of samples were used for training and prediction.

### Small sample-based prediction

We wanted to estimate the number of samples needed to build a reliable Random Forest classifier. A subset of 2, 5, 10, or 20 random samples was used for allelic prediction and 10 or 20 samples were used for QTL prediction. The sampling was repeated 10 times to generate 10 such subpanels, for each of which an ROC curve was drawn based on 10 Random Forest classifiers resulted from a 5-fold cross validation with two repetitions each time. The variability among the 10 ROC curves from the 10 subpanels was observed. Next, we sought to apply our model for predicting missing positive pairs from small-size panels. To this end, we selected untestable cases in which we could not assess allelic imbalance due to absence of heterozygotes among the given samples. In some cases, observation could not be made because we filtered out heterozygous SNPs according to the read depth and imbalance ratio as described above. The previously built Random Forest classifiers for 10 samples were used to rescue missing positive cases from the untestable set. As described above, the sampling was repeated 10 times each with 10 Random Forest classifiers. Therefore, an untestable set was generated for each of the 10 subsets, and the matched 10 classifiers were run for each target set. Positive calls were identified as being supported by more than 5 of the 10 classifiers. To assess the capability of our prediction method, we examined how many of the positive calls actually displayed allelic imbalance when the whole set of samples in the panel was tested for allele specificity.

## Supporting information

S1 Fig Reduced explanatory power due to chromatin randomization. The chromatin interactome data were merged and permuted to connect allele imbalance pairs randomly. Because only allele-specific pairs in the same regulatory direction were mapped, a certain level of explanatory power was achieved even with randomization. However, there was an overall reduction compared to the real data (compare the grey curves with the coloured lines). Four different permutations were performed for H3K27ac.
(PDF)

S2 Fig Allelic mapping for RASSF5. The ChIP-seq SNPs (blue) showing allele imbalance (blue bar graphs) with the risk allele underrepresented were connected to RASSF5 as indicated by different chromatin interactome datasets (black lines). The RNA-seq variants (red) showed allele-specific expression (red bar graphs) in the same direction as the ChIP-seq variants. eQTL mapping failed to detect association (boxplots).
(PDF)

S3 Fig Prediction failure due to feature randomization. Random Forest prediction was performed after permuting the assignment of features to each pair. Permutation was repeated 10 times (grey ROC curves).
(PDF)

S1 Table Reference panel data. (XLSX)

S2 Table Immune-related diseases and traits. (XLSX)

S3 Table Reference chromatin interactome datasets. (XLSX)

S4 Table Gene ontology enrichment analysis. (XLSX)

S5 Table Features used for Random Forest. (XLSX)

S6 Table Variable importance of Random Forest for H3K27ac. (XLSX)

S7 Table Variable importance of Random Forest for H3K4me1. (XLSX)
