# Supplementary figures and images for: Predictive long-range allele-specific mapping of regulatory variants and target transcripts
Source: PLoS One. 2017 Apr 13;12(4):e0175768. doi: 10.1371/journal.pone.0175768 (PMC5391085; doi:10.1371/journal.pone.0175768)

S1 Fig

A

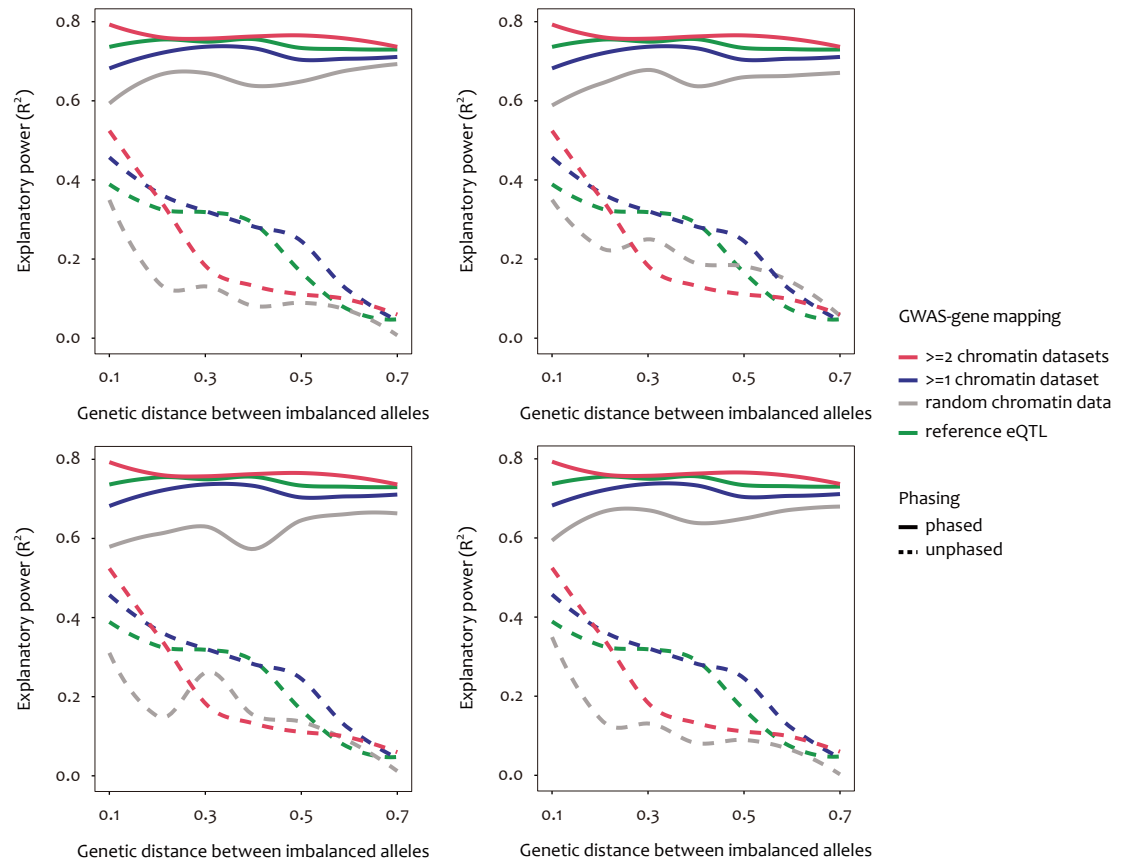

Supplement: S1 Fig — The chromatin interactome data were merged and permuted to connect allele imbalance pairs randomly. Because only allele-specific pairs in the same regulatory direction were mapped, a certain level of explanatory power was achieved even with randomization. However, there was an overall reduction compared to the real data (compare the grey curves with the coloured lines). Four different permutations were performed for H3K27ac. (PDF) [file pone.0175768.s001.pdf]

S2 Fig

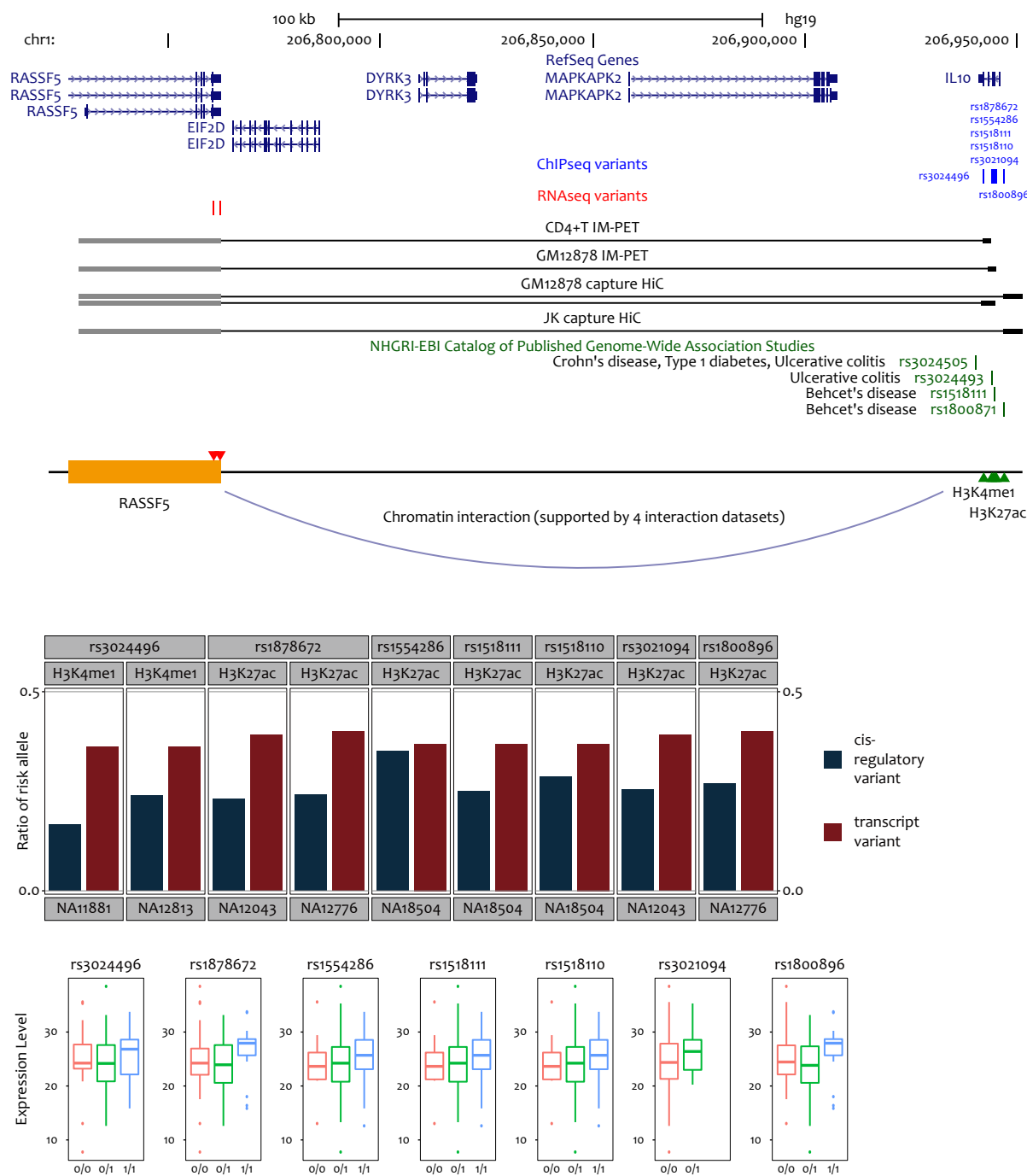

Supplement: S2 Fig — The ChIP-seq SNPs (blue) showing allele imbalance (blue bar graphs) with the risk allele underrepresented were connected to RASSF5 as indicated by different chromatin interactome datasets (black lines). The RNA-seq variants (red) showed allele-specific expression (red bar graphs) in the same direction as the ChIP-seq variants. eQTL mapping failed to detect association (boxplots). (PDF) [file pone.0175768.s002.pdf]

S3 Fig

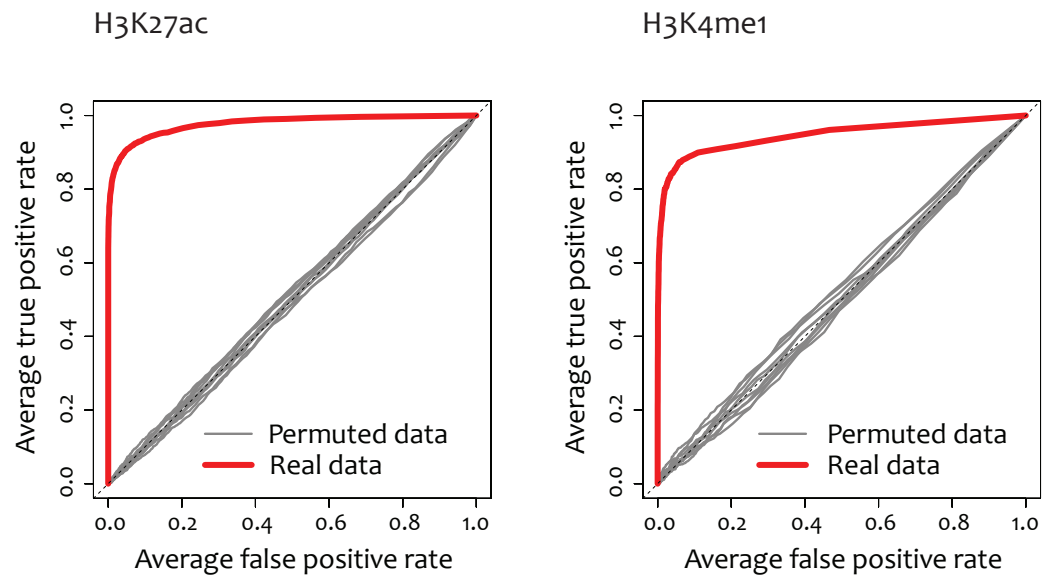

Supplement: S3 Fig — Random Forest prediction was performed after permuting the assignment of features to each pair. Permutation was repeated 10 times (grey ROC curves). (PDF) [file pone.0175768.s003.pdf]
